# Supplementary material for: Pathological and genetic aspects of spontaneous mammary gland tumor in Tupaia belangeri (tree shrew)
Source: PLoS One. 2020 May 18;15(5):e0233232. doi: 10.1371/journal.pone.0233232 (PMC7233572; doi:10.1371/journal.pone.0233232)

■ Measurement of fatty liver in tree shrew

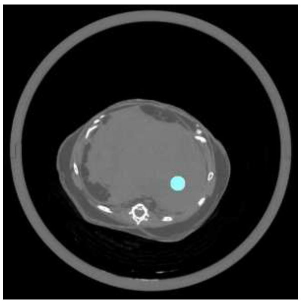

Fat content; 4.6 %

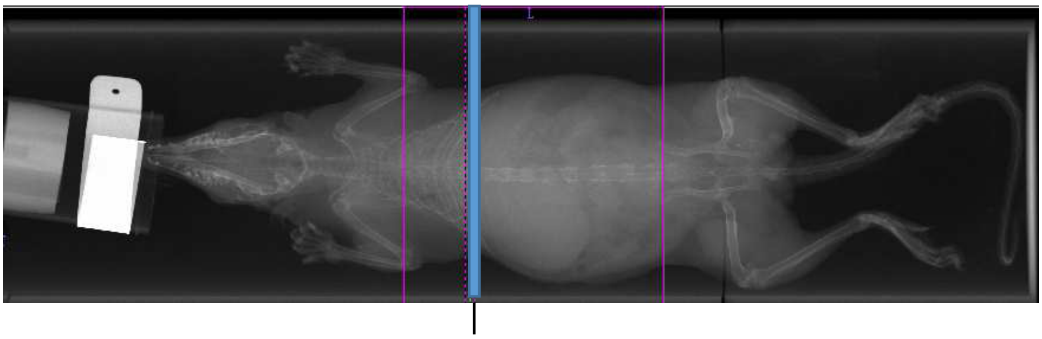

analysis
area

■ Measurement of liver volume in tree shrew

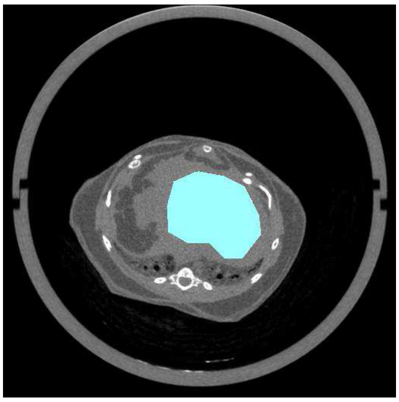

Average CT value; 18.2 (HU)

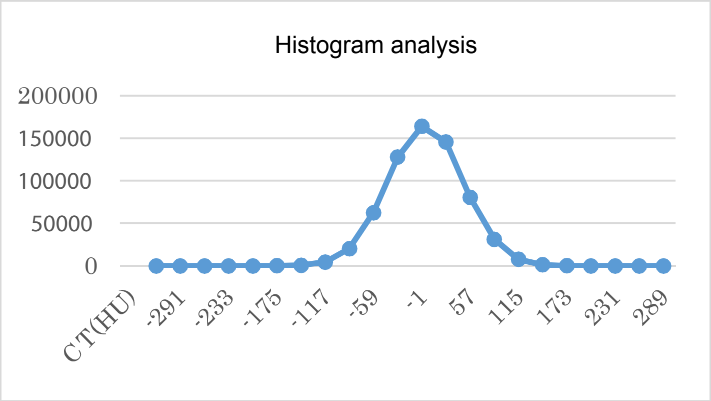

■ Measurement of fat ratio in tree shrew

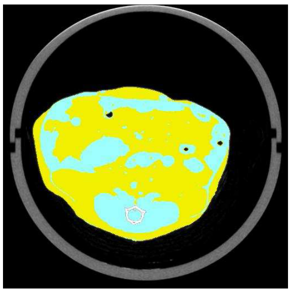

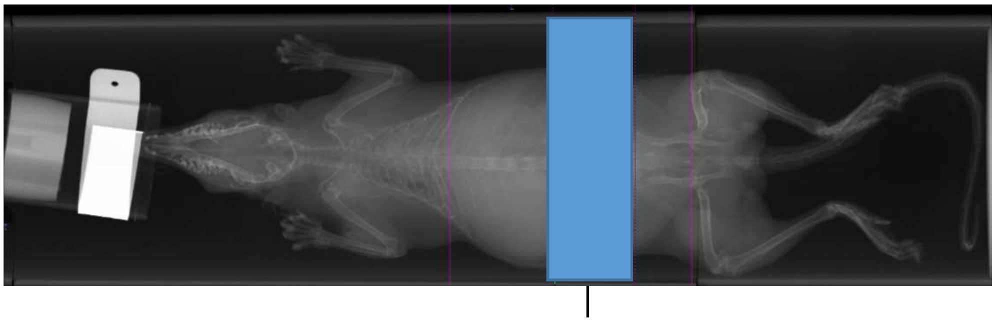

analysis
area

Fat ratio; 55.637 %

■ Measurement of brain volume and analysis of foreign materials (blue or red) in tree shrew

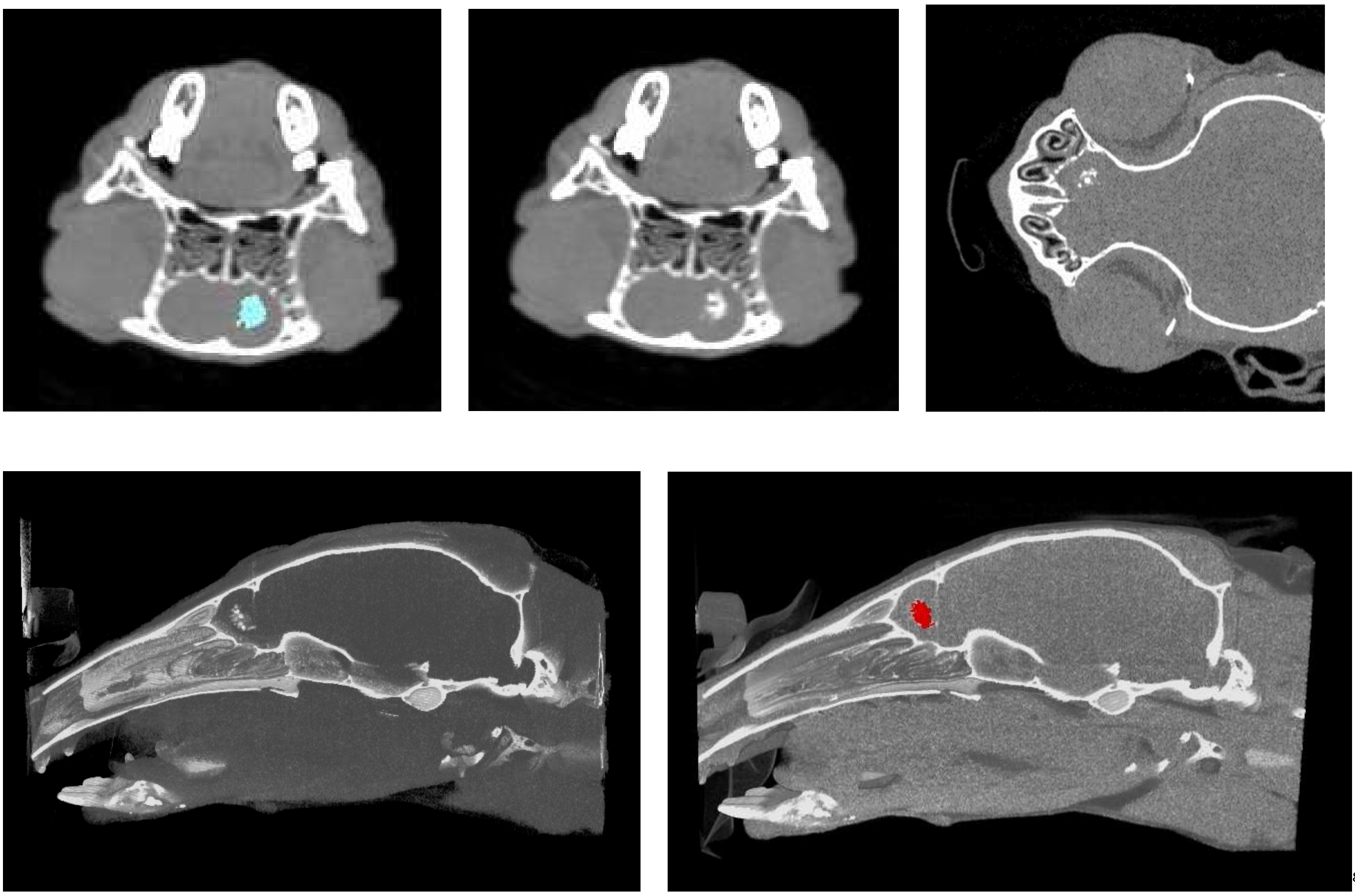

Supplement: S2 Data — (PDF) [file pone.0233232.s002.pdf]
