# Supplementary figures and images for: Pathological and genetic aspects of spontaneous mammary gland tumor in Tupaia belangeri (tree shrew)
Source: PLoS One. 2020 May 18;15(5):e0233232. doi: 10.1371/journal.pone.0233232 (PMC7233572; doi:10.1371/journal.pone.0233232)

(A)

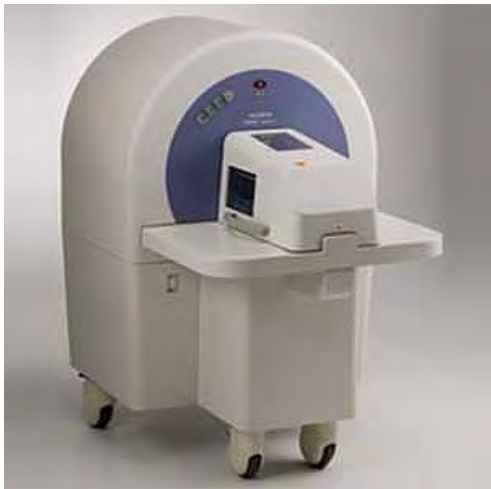

(B)

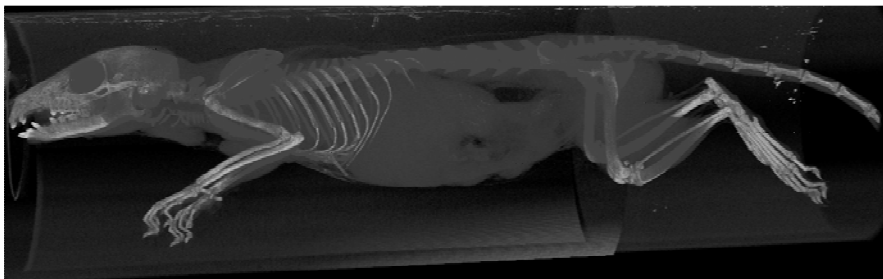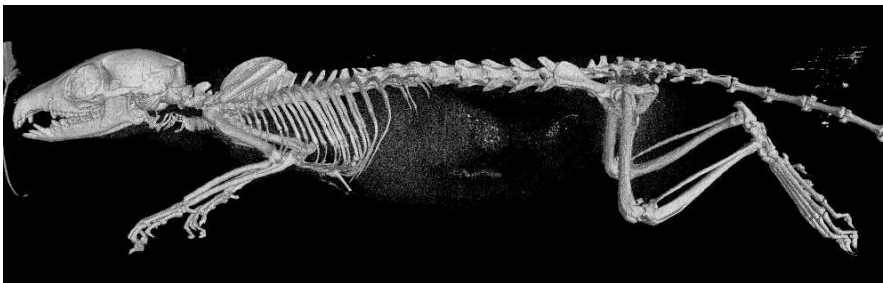

(C)

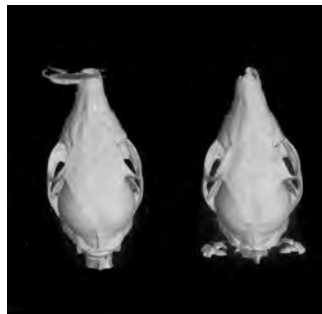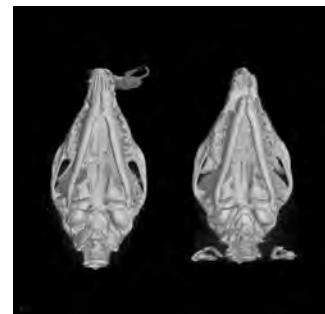

(上が♀、下が♂)

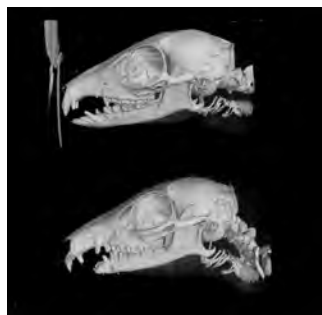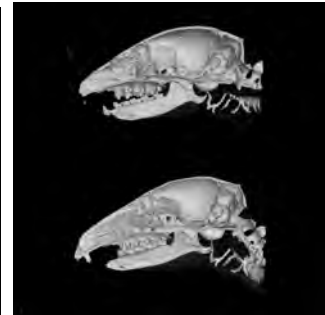

Fig.S1

Supplement: S1 Fig — (A) Outlook of CT LCT-200 (Hitachi Ltd., Tokyo Japan). (B) Whole body structure (upper) and bone structure (lower). (C) The skull structure of tree shrew (#26, 4 years 11 months). (PDF) [file pone.0233232.s003.pdf]

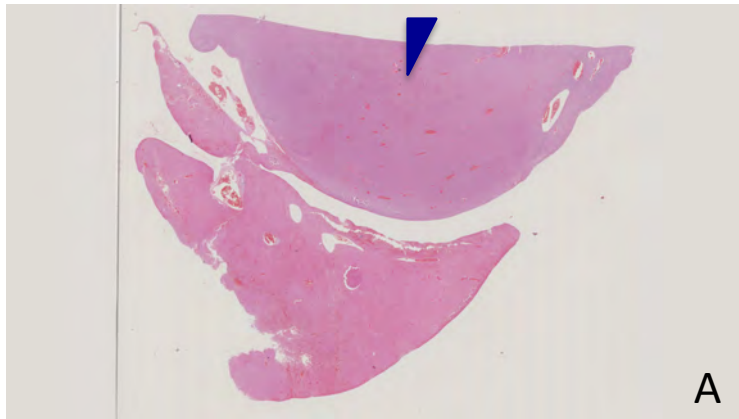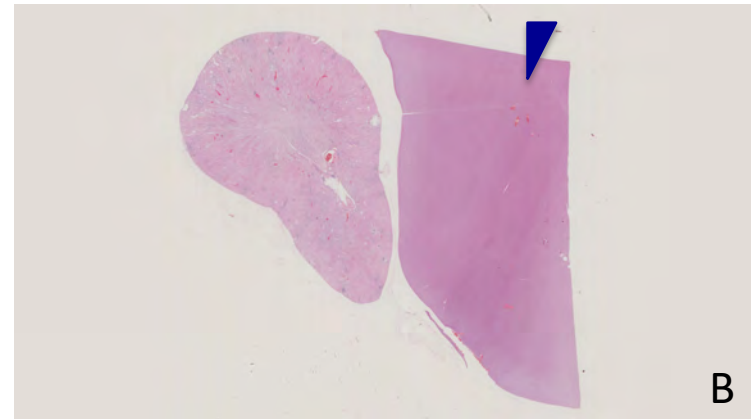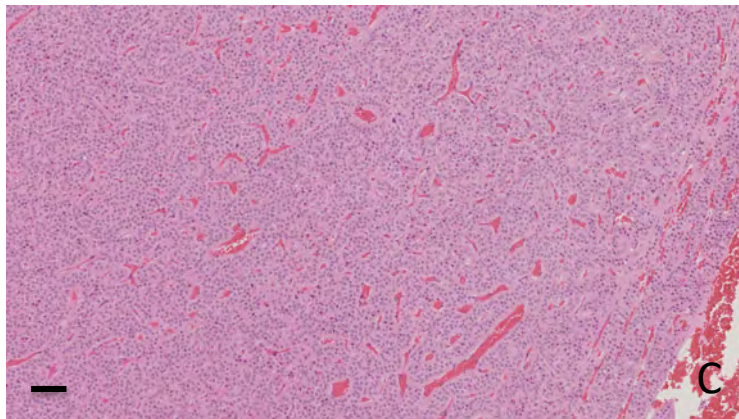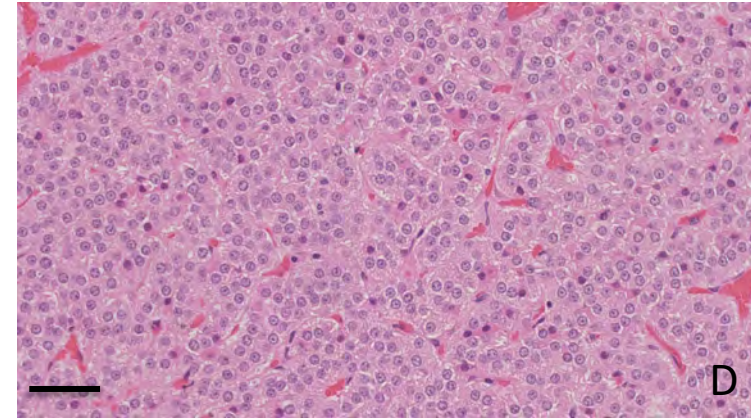

**Fig.S2**

Supplement: S2 Fig — (A) Tumor (upper, blue arrow) and liver (lower). (B) Tumor (blue arrow) and kidney (left). (C) Tumor (x40) (D) Tumor (x200), Bar-100μm. (PDF) [file pone.0233232.s004.pdf]
