# Supplementary material for: Pathological and genetic aspects of spontaneous mammary gland tumor in Tupaia belangeri (tree shrew)
Source: PLoS One. 2020 May 18;15(5):e0233232. doi: 10.1371/journal.pone.0233232 (PMC7233572; doi:10.1371/journal.pone.0233232)
Supplement: S1 Table — (DOCX) [file pone.0233232.s005.docx]

**Table S1** J-score

| J-score | Evaluation | Ratio of positive cells |
| --- | --- | --- |
| 3b | High expression | >50% |
| 3a | Positive | More than 10%, less than 50 % |
| 2 | Marginal area | More than 1 %, less than 10 % |
| 1 | Marginal area | Less than 1 % |
| 0 | Negative | negative |
