# Supplementary material for: Pathological and genetic aspects of spontaneous mammary gland tumor in Tupaia belangeri (tree shrew)
Source: PLoS One. 2020 May 18;15(5):e0233232. doi: 10.1371/journal.pone.0233232 (PMC7233572; doi:10.1371/journal.pone.0233232)
Supplement: S2 Table — (DOCX) [file pone.0233232.s006.docx]

**Table S2** HER-2 score

| Score | Remark | Staining pattern |
| --- | --- | --- |
| 0 | Negative | No stained cells, or less than 10% weakly stained cells |
| 1+ | Negative | More than 10 cells were weakly stained |
| 2+ | Positive | More than 10 % cells or membrane were stained  Less than 10 % cells or membrane were strongly stained |
| 3+ | Positive | More than 10 % cells or membrane were strongly stained |
