# Supplementary material for: Pathological and genetic aspects of spontaneous mammary gland tumor in Tupaia belangeri (tree shrew)
Source: PLoS One. 2020 May 18;15(5):e0233232. doi: 10.1371/journal.pone.0233232 (PMC7233572; doi:10.1371/journal.pone.0233232)
Supplement: S1 File — (DOCX) [file pone.0233232.s007.docx]

**Supplementary methods**

**Evaluation of malignancy**

Atypical morphology of the nucleus and cytoplasm, clear invasion, and metastasis to lymph nodes and other tissues were judged as malignant characters.

**Evaluation of ER, PR positive cells**

After the staining of tumor tissues, we selected more than 5 areas at lower magnification (x40) and counted more than 1,000 tumor cells in higher magnification (x400), following J-score (Table S1) [1]. In the boundary area, “more than 1%” was judged to be positive [2].

**Evaluation of Ki-67 positive**

Distribution of positive cells (from among >1,000 tumor cells) were evaluated under lower magnification (x40) and under higher magnification (x400) in at least 2 areas, viz., in hot spot and cancerous area. Ratio of positive cells to counted cells are indicated as labeling index (LI) [3].

**Evaluation of HER-2 positive**

Evaluation was followed by the Hercep Test (DAKO). Scoring was performed as shown in Table S2.

**References**

[1] Kurosumi, M., Immunohistochemical assessment of hormone receptor status using new scoring system (J-score) in breast cancer. Breast Cancer 14 189-193, 2007.

[2] Pathologists’ Guidline Reccomendations for Immunohistochemical Testing of Estrogen and Progesterone Receptors in Breast Cancer (ASCO/CAP guidline).

[3] Dowsett, M., et al., Assesment of Ki67 in Breast Cancer: Recommendations from the international Ki67 in breast cancer working group. J.Natl. Cancer Inst. 103 1656-1664, 2011.
